# SARS-CoV-2 Attacks in the Brain: Focus on the Sialome

**DOI:** 10.3390/cells11091458

**Published:** 2022-04-26

**Authors:** Przemyslaw Wielgat, Karolina Narejko, Halina Car

**Affiliations:** 1 Department of Clinical Pharmacology, Medical University of Bialystok, Waszyngtona 15A, 15-274 Bialystok, Poland; knarejko1@student.umb.edu.pl (K.N.); halina.car@umb.edu.pl (H.C.); 2 Department of Experimental Pharmacology, Medical University of Bialystok, Szpitalna 37, 15-265 Bialystok, Poland

**Keywords:** SARS-CoV-2, sialic acid, COVID-19, brain, neurons, inflammation

## Abstract

The epidemiological observations suggest that respiratory and gastrointestinal symptoms caused by severe acute respiratory coronavirus 2 (SARS-CoV-2) are accompanied by short- and long-term neurological manifestations. There is increasing evidence that the neuroinvasive potential of SARS-CoV-2 is closely related to its capacity to interact with cell membrane sialome. Given the wide expression of sialylated compounds of cell membranes in the brain, the interplay between cell membrane sialoglycans and the virus is crucial for its attachment and cell entry, transport, neuronal damage and brain immunity. Here, we focus on the significance of the brain sialome in the progress of coronavirus disease 2019 (COVID-19) and SARS-CoV-2-induced neuropathology.

## 1. Introduction

Coronavirus disease 2019 (COVID-19) is a highly heterogenic and complex disorder with an aberrant immune response to the infection with human coronavirus, known as SARS-CoV-2 (severe acute respiratory syndrome coronavirus 2) [1]. The extensive clinical observations in the field of diagnostics, therapeutic management and prevention have shown that COVID-19 displays a wide range of severity, from asymptomatic to fatal clinical outcomes. In the acute phase of infection, SARS-CoV-2 predominantly affects lung function through invasion and replication in the upper respiratory tract, and then fast spreading in the lower respiratory tracts resulting in pneumonia of severe or fatal course. However, a range of symptoms persist for a long time after infection and correspond to the effects of SARS-CoV-2 on multiorgan systems. According to the National Institute for Health and Care Excellence (NICE) guidelines and reports of the British Office for National Statistics (ONS) the post-COVID-19 conditions within the pulmonary, cardiovascular and nervous system, routinely called long-COVID, have been observed in more than 20% of individuals for several weeks following acute SARS-CoV-2 infection [2,3]. The high biodiversity and infectious potential are determined by the sets of mutations that result in variants of specific transmissibility and antigenicity. Based on SARS-CoV-2 molecular structure and genetic heterogeneity, five “variants of concern” (VoC) have been characterized according to their infectious potential, spread and fatality rate [4,5]. These characteristics depend on the alterations in viral structural proteins, including envelope (E), membrane (M), nucleocapsid (N), and the spike protein (S) that coordinate replication and viral assembly. In the aspect of viral biology, the diversity of these proteins results from the high rate of genetic variations and determinates viral pathogenicity, infectivity, and antigenicity [6]. Since the COVID-19 pandemic’s onset, scientists have been trying to define the complete mechanism that allows the viral particles to enter and infect healthy human cells. SARS-CoV-2, like other β-coronaviruses (β-CoVs), exploits the S protein to initiate the interaction between viral capsid and the host cell membranes. The mechanisms underlying the entry into the host cells recruit the functional domains of S protein that recognize specific host receptors and mediate the fusion with host cell membranes. This process starts from the activation of membrane fusion activity through the cleavage of S protein into S1 and S2 domains mediated by the host cellular proteases, including transmembrane protease serine 2 (TMPRSS2), furin, and cathepsins [7]. Proteolytic processing within the S1 subunit is crucial for recognition and interaction between its receptor-binding domain (RBD) and the host receptors [8]. Both clinical and experimental studies have identified the angiotensin-converting enzyme 2 (ACE2) as the entry receptor for SARS-CoV-2 into the human cells [9,10,11]. As has been shown, the expression of transmembrane, but not soluble ACE2 isoform was positively correlated with viral RNA load and TMPRSS2 expression depending on the age and biological sex of the host [12]. In addition, the risk of infection of SARS-CoV-2 and promotion of its replication can be promoted by renin angiotensin system (RAS) modulators, including angiotensin receptors blockers (ARB), as has been demonstrated in cultured Vero E6 cells [13].

The variation in disease severity after SARS-CoV-2 infection is dictated by the strong cellular tropism and the broad expression of ACE2 in several organs of the body, and is thereby closely linked to progressive dysfunction within the immune, pulmonary, cardiovascular and nervous systems [14]. However, there is increasing evidence on the recruitment of the highly sialylated cellular glycoconjugates in viral entry, kinetic and immune regulation in SARS-CoV-2 infections. Since the structural analysis of the N-terminal domain (NTD) of SARS-CoV-2 has reported the ability of specific amino acid sequences to form sialoside binding pockets, the interaction with the host surface glycocalyx can be functionally involved in the rapid infectivity and spread in sialic acid-rich organs [15,16]. The comparable analysis of human β-CoVs in the field of mechanisms of infection, tropism, and pathogenesis revealed the role of sialic acids as regulators of SARS-CoV-2’s infective potential and invasiveness [17,18]. This review briefly focuses on the importance of sialic acids in the SARS-CoV-2-related pathology in the central nervous system. We present the sialoglycans as an alternative viral entry mechanism and discuss how human cell surface sialome has been affected by COVID-19 and corresponds to clinical disturbances within the brain.

## 2. Sialome of the Brain—The Regulatory System of Structure and Function

Sialome is a cellular repertoire of sialic acid that forms a diverse subtype of the glycome [19]. The human cell surfaces are covered with a dense network of glycans that are covalently attached to macromolecules to form glycoconjugates. Glycosylation of proteins and lipids is one of the most critical molecular modifications that modulates their conformation, structural stability and turnover [20]. Both proteins and lipids undergo glycosylation in the Golgi apparatus and endoplasmic reticulum, where 17 different monosaccharides can be attached under the control of approximately 200 glycosyltransferases. In result, the mammalian glycome is characterized by highly diverse organization composed of at least 7000 different glycan structures [21]. Structural studies of cell membranes have shown that glycoconjugates are mostly decorated by sialic acid at the non-reducing end of the glycan chain. The family of sialic acids includes nine carbon backbone ketosugars characterised by high diversity due to their chemical modifications, including methylation, acetylation, sulfation and phosphorylation. These chemical derivatives can be attached to terminal sugar structures via α2.3, α2.6 and α2.8 linkage in glycoproteins and glycolipids and thereby dictate the negative charge and hydrophilicity of the cell surface [20]. The family of human glycolipids is dominated by four major gangliosides, GM1, GD1a, GD1b and GT1b, that carry 75% of the total brain’s sialic acids, whereas the remaining 25% are incorporated in the highly diverse sialoglycoproteins. The transfer of sialic acid residues is catalysed by 20 different sialyltransferases that have been identified in both human and murine cells. In the brain, the morphological reorganisation of the neuronal network is closely related to the function of polysialyltransferases, including ST8SiaII and ST8SiaIV that are implicated in posttranslational modification of neuronal cell adhesion molecules (NCAMs) in young and adult neurons [22,23]. Given a wide organization of oligosaccharide structures and their chemical and physical features, sialylated glycoconjugates seem to be the main players in the regulation of cellular biology. The strong hydrophilicity, negative charge and location on the extracellular surface determine the predominant function in cell–cell recognition and interplay between cells and elements of their surrounding environment [24,25,26]. In the brain, both N- and O-glycosidically linked oligosaccharide donors are attached to proteins and lipids known to be involved in the cell development and differentiation, and function of the nervous system. The previous advances in glycobiology and neurobiology have shown that the linkage of glycan chains with protein or lipid core affects neurite outgrowth, axon pathfinding and synaptogenesis by modulating the structure, stability and interaction abilities of neuronal macromolecules. As shown, the decoration of adult neuronal cell surfaces by long homopolymers of α2.8-sialic acids, called polysialic acid (PSA), is restricted to the regions featured by intense axonal sprouting and cell migration that underlie the mechanisms of stimuli learning and memory formation [27,28]. The accumulating data suggest that qualitative and quantitative changes in glycan epitopes result in impaired cell adhesion, synaptic plasticity and regeneration that underlie neurological deficits. Moreover, the sialic acid-induced reduction of mutual adhesiveness promotes the migration of hypersialylated cancer cells in the metastatic process [29,30]. According to the clinical studies, sialic acid-containing glycoconjugates, including GD2, GD3 and PSA–NCAM, are highly expressed in high-grade brain tumours and enable their increased invasion and mobility [31,32,33]. In the field of the high functions of the brain, neuronal glycoconjugates regulate synaptic neurotransmission via modulation of proteins involved in neurotransmitter release and transport, as well as regulation of ion channels that control membrane excitability and response to milieu stimuli. Due to the negative charge of sialic acid-created cell membranes, calcium ions are tightly bound by synaptic sialylated glycolipids and act as neuromodulators of neurotransmitters release from synaptic vesicles [34]. In addition, it has been shown that glycolipids, as the main components of cell surface glycocalyx, play a pivotal role in the regeneration process due to their pro-survival effects and control of myelin sheath generation. The membrane GD1a and GD1b gangliosides promote the interaction with the myelin-associated glycoprotein (MAG) via NeuAc2–3Gal1–3GalNAc saccharide sequence and thereby facilitate the axon myelination but not the regeneration of injured neurons. It may reflect the dual function of sialic acids in the recovery processes within the brain [20,35].

Finally, the glycosylation of the cell surface is critical in biological recognition. The cellular systems of regulatory proteins called immune checkpoints recruit stimulatory and inhibitory molecules that interact with their specific ligands and control self-tolerance processes to avoid immune injury. It is of particular importance in tissue homeostasis maintenance and preventing autoimmune reactions against self-produced antigens through the self-tolerance processes [36]. Given the glycosylation pattern and branching architecture, sialoglycans are an inherent part of the complex mechanism of regulation of the innate and adaptive immune response. Thus, cellular sialoglycans can be considered as self-associated molecular patterns (SAMPs) which are recognized by inhibitory receptors, to maintain the non-activated state of immune cells and dampen their reactivity following an immune response. In relation to the host cells and SAMPs, pathogens present molecular systems (PAMPs, pathogen-associated molecular patterns) that are recognized by the host receptors and initiate the immune response. The interplay between PAMPs and pathogen recognition receptors (PRRs) of the host is characterized by high recognition ability and results in the overresponse of the host defence mechanisms [37]. At the level of cellular and molecular processes, sialome constitutes the ligands for sialoglycan-binding proteins, such as the selectins, and the sialic acid-binding immunoglobulin-like lectins (Siglecs) that trigger activatory and inhibitory signals and regulate the secretory activity and turnover of immune cells. In viral and bacterial invasion, the interplay between sialic acid-expressing pathogens and the host sialic acid-binding receptors has been defined as a crucial part of the biological recognition and defence against invaders. However, the surface of human pathogens can be covered by analogous glycan epitopes that mimic the host’s glycosylation patterns, resulting in immune evasion. The clinical and experimental observations suggest the engagement of the Siglec–sialic acid axis in the molecular mimicry and self-nonself discrimination by the innate immune system that underlie multiple pathological processes [37].

## 3. SARS-CoV-2 in the Brain—Destination, Route and Effects

Although the neurological symptoms of COVID-19 have been widely described, the pathogenesis of SARS-CoV-2 infection in the CNS is still poorly understood. Both human autopsy reports and animal models have shown evidence of the brain as a target organ for SARS-CoV-2 infection that closely correlates with neurodegenerative alterations and contributes to high morbidity and mortality ratio [38,39]. The analysis of spatiotemporal dynamics of SARS-CoV-2 infection in the lethal mouse model of COVID-19 revealed the relationship between viral neuroinvasion and direct injury of the brain and spinal neurons. The experimental observations by Kumari et al. confirmed high levels of virus replication in the brains of transgenic K18-hACE2 mice after intranasal inoculation [39]. Data for several brain sections in mice, including the hippocampus, cortex and cerebellum, showed that the level of SARS-CoV-2 antigen was significantly higher than its expression in the lungs. The restricted neurotropism was accompanied by infiltration of leukocytes and elevated expression of cytokines and chemokines at gene and protein levels [39]. The case series of autopsies from patients who died from COVID-19 revealed multiple neuropathological changes and correlation with peak viral level, activation of microglia, infiltration by cytotoxic T cells and astrogliosis [38]. In mice, the virus-induced changes in brain immunity can cause extensive neuroinflammation and contribute to multiple brain dysfunctions, whereas in the lungs the viral particles undergo clearance and symptoms of respiratory illness start to decline. This finding reflects the long consequences of COVID-19 in humans. Indeed, the expression of human ACE2 in tested transgenic animals and its distribution in the human brain suggests the viral entry via ACE2-expressing olfactory nerves or ACE2-expressing endothelial cells of the blood–brain barrier (BBB) and then migration across neuroepithelium to invade the CNS [40]. Despite the hypothesis for the mechanism of SARS-CoV-2 invasion being confirmed by analysis of ACE2 tissue distribution, the long-term neurological consequences seem to be dependent on alternative mechanisms of neuroinvasion [41]. As an investigation by Carrossino et al. indicates, SARS-CoV-2 tropism in the brain is not limited to the ACE2 positive cells whereas the broad expression of ACE2 in the brain capillaries is also not associated with viral antigen coexpression [42]. Molecular mechanisms that underlie the viral attachment and entry lead to a wide range of symptoms, ranging from mild neurological manifestations to severe form with high risk of lethal outcomes [43]. The influence of SARS-CoV-2 on brain tissue depends on the direct and indirect mechanisms that disturb neuronal functions including signal transmission and viability [44]. The post mortem examination of frontal lobe brain sections revealed the existence of multiple viral particles located within vesicles in the cytoplasm of a neuronal soma, which was correlated with neuronal damage and apoptosis [45]. Tiwari et al. demonstrated that these changes were accompanied by enhanced caspase-3 gene transcription, whereas the antiapoptotic genes, including *BCL2* and *BAX*, were downregulated [46]. The direct damage to neurons has been considered as the main mechanism of early symptoms of COVID-19. Given the importance of mucus membrane in the nose and mouth in viral deposition and transmission, the pathology of olfactory and gustatory sensory neurons is one of the most frequent neurological events during SARS-CoV-2 infection. Additionally, direct damage to sensory neurons by virus particles coexists with an altered immune response in nervous tissue and results in olfactory and gustatory disturbances that have been reported in up to 88% of individuals and routinely used as mass screening tools for COVID-19 [47]. According to the preliminary hypothesis by Islam et al., fragments of SARS-CoV-2 proteome present amyloidogenic potential and exert neurotoxic effects against neuronal cell lines routinely used in cellular neurodegeneration in vitro models [48]. This direct mechanism of toxicity and neuronal damage may explain some of the long-term events, including long-term anosmia and ageusia. Independent clinical and experimental studies have revealed that the mechanisms underlying neurological dysfunction are closely related to the role played by the immune system and aberrant inflammatory response in the host cells [49,50]. The dysregulation in the balance between production and release of proinflammatory and anti-inflammatory factors results in systemic and/or diffuse hyperinflammatory states, usually called cytokine storms and macrophage activation syndrome, respectively. It has been found that innate leukocytes, including neutrophils and monocytes, act as the main protectors against SARS-CoV-2 invasion; however, this function can be modulated by the action of Th1 and Th17 effector cells [51]. In the brain, infiltrating T17 cells belong to the main mediators of cytokine storm. The secretory activity of these cells results in the high levels of IL-1β, IL-2, IL-8, IL-9, IL-17, TNFα and CXCL-10 that penetrate the BBB and activate the CNS resident macrophages and astrocytes to produce neuronal cytokines and thereby induce neural dysfunction [52,53,54]. An excessive increase in the expression of proinflammatory factors in the brain leads to microglial activation and proliferation, which disturb the immune homeostasis between neurons, non-neuronal and immune cells. As shown, the activation of microglial cells results in high inflammatory mediators that promote upregulation of neuronal expression of NMDA receptors and glutamate and thereby excitotoxicity and neuronal death [52,55]. Microglia-induced oxidative stress refers to increased levels of reactive oxygen species (ROS) that contribute to the loss of cerebral endothelial tight junctions and thereby modulate innate and peripheral adaptive immunity in the brain. It has been found that the count of peripheral CD4+ and CD8+ T cells is correlated with the severe clinical course of infection. In the context of neurological deficits, an increased level of activated CD4+ and CD8+ cells in the brain coexists with damage to the myelin layer and the loss of axons [56]. Neurodegeneration and demyelination are less frequent and more health-threatening neurological manifestations; however, changes in laboratory markers and clinical symptoms of Guillain-Barre syndrome, cerebellar ataxia, inflammatory demyelinating polyneuropathy and inflammatory vasculopathy have been observed several days after SARS-CoV-2 infection [47,57]. Besides the broad alterations in the immune status of both infiltrated and resident immune cells, SARS-CoV-2 causes persistent inflammation and brain injury via the altered immune activity of the brain cells. In the heterogeneous population, neurons showed the highest level of viral RNA that was accompanied by the upregulation of genes involved in brain immunity, including *IL6*, *TLR3*, *TLR7*, *IFIT3*, *OAS2* and *CDK5* [46]. At the protein level, enhanced expression of IL-6 was detected in sensory neurons in the olfactory bulbs. Given the importance of IL-6 in the immune pathways and cytokine storm formation, the loss of smell is closely linked to IL-6 mediated neuronal degradation, whereas the reconditioning of olfaction is associated with reduced IL-6 expression [58]. Additionally, high levels of IL-1β, IL-6 and TNFα in the brain have been described as negative predictive factors of cognitive, psychomotor and neurovegetative symptoms [59]. In the field of brain anatomy, these changes were correlated with structural alterations in the functional regions due to decreased neurogenesis and induced apoptosis [60]. The neuronal loss results in brain shrinkage; however, the inflammatory processes promote the swellings and increase the volume of the pathology-related regions. It is of particular importance in brain biology and contributes to alterations in synaptic plasticity, impairment of neurotransmitters turnover, and in consequence, atrophy of neuronal tissue. The analysis of structural MRI brain scans of SARS-CoV-2 infected patients obtained from Biobank UK revealed a greater reduction in global brain size, including grey matter thickness, and alterations in markers of tissue damage in functionally active regions. It has been speculated that the time-dependent structural alterations in the parahippocampal gyrus and hippocampus reflect the risk of the development of features of Alzheimer’s disease (AD) as a possible long-term consequence of SARS-CoV-2 [61]. In relation to COVID-19, the loss of dopaminergic neurons in substantial nigra has been defined as a strong risk factor of Parkinsonism and depression, whereas the reduced volume of the hippocampus and impaired neuroplasticity underlie cognitive deficits [62,63]. According to Lipton et al., effective neurotransmission requires at least 30% cerebral oxygen delivery and is strongly interrupted by hypoxia [64]. Neurons, being especially vulnerable to low oxygen, become dysfunctional because of impaired mitochondrial function, Krebs cycle inhibition, decreased levels of adenosine triphosphate (ATP) and functional changes in oxygen-sensitive Na^+^ and K^+^ ion channels. Besides the metabolic effects, hypoxia promotes the upregulation of pro-inflammatory cytokines associated with neural damage [65,66,67]. These processes contribute to the complex of cognitive dysfunction, including memory problems, lack of mental clarity, poor concentration and inability to focus, which have been customarily described as “brain fog” [68]. Several studies, including systemic reviews and meta-analyses, have been focused on sleep disturbances associated with the SARS-CoV-2 infection. According to the observation by Pataka et al., the prevalence of sleep problems was observed in 57% of patients diagnosed with COVID-19 [69]. Despite COVID-19-induced insomnia being classified as a long-term clinical complication, the mechanisms underlying this phenomenon are still poorly understood. The recent findings in this field suggest that sleep impairment depends on the multifunctional mechanism that includes stress exposure, immunological reactions, the severity of the viral infection and the effects of applied therapies. Interestingly, the involvement of SARS-CoV-2-associated neuroinflammation seems to be the crucial factor implicated in the overall pathological processes observed in the CNS during infection. It has been shown that sleep disorders can induce BBB leakage via neuroinflammation, which contributes to the long-COVID related phenomenon. There is emerging evidence that sleep apnoea in COVID-19 patients induces systemic low-grade inflammation featured by the release of pro-inflammatory and immune-derived inflammatory mediators that disrupt endothelial junctions in the hippocampus, cause penetration of the BBB and decrease the complexity of interendothelial junctions [70]. Therefore, COVID-induced sleep impairment can be classified as an additional factor that potentiates the development of CNS hyperinflammation.

## 4. Sialic Acid—One of Unknowns of Neuropathogenesis of SARS-CoV-2 Infection?

Since the role of sialoglycans has been described in physiological homeostasis and the clinical course of pathologies, they are extensively examined as players in the mechanism of pathogen invasion, clinical manifestations and potential targeted therapy. The advances in the field of microbiology highlight the functional importance of sialic acid in the mechanism of viral attachment and the cellular entrance, interacting with bacterial toxins and evasion of host immunity [71]. Therefore, the analysis of the sialylation pattern of both serum and cell membrane proteins and lipids can be used as a predictive marker of invasion and related pathologies. Based on colorimetric, fluorometric and enzymatic methods, the multiple novel sialic acid-related biomarkers have been described in the clinical analysis of human tissues [72]. Given the high concentration of sialic acids in the brain and their importance in the biology of neural tissue, the involvement of neural sialoglycans in recognizing SARS-CoV-2 can be crucial for its invasion within the CNS and clinical course of infection. It is of particular importance since the structural and functional changes in the CNS have been described as long-term complications of SARS-CoV-2 infection. The recruitment of immunological methods based on specific antibodies and labelled sialic acid-binding lectins allows assessment of the qualitative and quantitative changes in sialylation patterns in both in vitro and in vivo studies. However, the systematic analysis of glycan structure, including glycan sequences, specificity of branching and types of linkage, requires highly sensitive analytic methods that include structure complexity and heterogeneity, and low natural abundance of glycome. The glycomics of COVID-19-related brain pathology has not been fully developed, but previous studies in the field of microbiology suggest the usefulness of matrix-assisted laser desorption/ionization time-of-flight (MALDI-TOF) mass spectrometry. Indeed, the analysis of glycan structures in selected brain areas using MALDI-TOF MS revealed the importance of glycan alterations in depressive-like behaviours during infection with *Toxoplasma gondii* [73]. Finally, the clinical assessment of sialic acid-dependent immunity-controlling systems can add a new dimension to the field of diagnosis in individuals with CNS comorbidities in SARS-CoV-2 infections.

### 4.1. Sialic Acids—Boosters of SARS-CoV-2 Invasion in the Brain?

**Figure 1:**
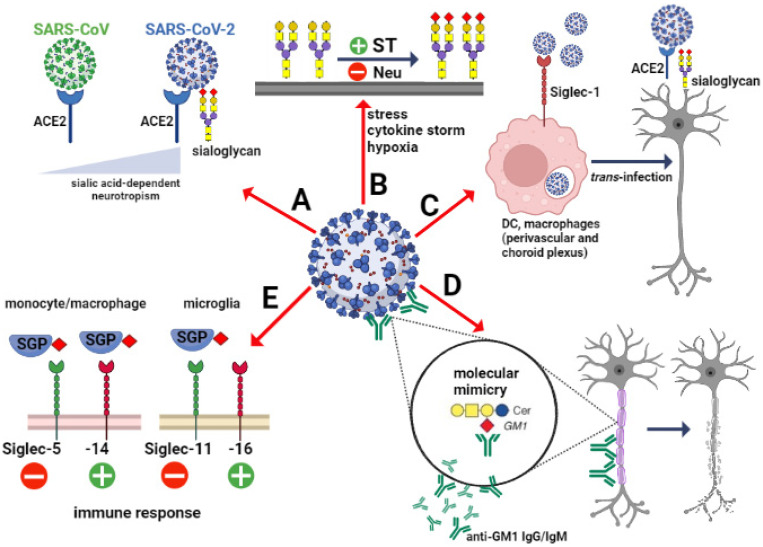
The engagement of sialic acid-dependent mechanisms in SARS-CoV-2 invasion in the human CNS. The ability to recognize and bind to the sialoglycans of the host determines the viral tropism to sialic acid-rich brain tissue (**A**). In response to viral infection-related hypoxia and cytokine storm, changes in the balance between sialyltransferases and neuraminidases lead to qualitative and quantitative alterations in cellular glycome (**B**). Siglec-1 on macrophages and dendritic cells interacts with sialylated glycans exposed on SARS-CoV-2, mediates its endocytosis and replication, and boosts viral transmission to neurons (**C**). The structural identity of sialoglycans expressed on the virus and host cells leads to neuronal degeneration as a result of the autoimmune process (**D**). The SARS-CoV-2 envelope sialylated glycans and sialylated secreted glycoproteins interact with Siglecs on the host cells, resulting in immune responses of different intensity (**E**); ACE2—Angiotensin-converting enzyme-2, ST—Sialyltransferases, Neu—Neuraminidases, DC—Dendritic cells, SGP—Sialylated secreted glycoprotein, GM1—Ganglioside GM1.

The importance of sialic acids in SARS-CoV-2 invasion depends on scenarios that include various types of cells involved in the mechanism of virus attachment and cell entry in the brain. As mentioned previously, the ACE2-expressing epithelial cells of the respiratory and gastrointestinal tract are the main target for SARS-CoV-2. However, the expression of ACE2 in the brain is closely restricted to several regions and types of cells. Analysis of the cell-type distribution of ACE2 confirmed its strong expression in endothelial and epithelial cells as well as inhibitory and excitatory neurons in substantia nigra [74,75]. Interestingly, no or few ACE2-expressing cells were found in the prefrontal cortex and hippocampus, whereas sialic acid concentration was enhanced and correlated with expression of SARS-CoV-2 particles [76]. This may confirm the overall hypothesis that the viral entry into CNS depends mainly on ACE2 protein in the olfactory neuroepithelium, vascular endothelial cells and pericytes, whereas the viral spreading to some brain areas can be associated with the sialome [77]. The affinity to sialoglycans in the brain can be a crucial factor that determines viral neurotropism (Figure 1A).

As shown, the several members of beta-coronaviruses demonstrate neurotropism; however, the mechanisms that promote their presence in the CNS are still poorly understood. Differences in the viral neurotropic potential can be explained by variations in the structure of the S protein, despite structural similarities.

SARS-CoV-2 binds to the ACE2 with 10–20-fold higher affinity than SARS-CoV [78]. This phenomenon closely correlates with the capacity to recognize and bind sialic acid and the presence of glycan-binding domain (GBD) in the S1 subunit [79,80]. Thus, the sialic acid-binding capacity of SARS-CoV-2 facilitates the interaction with the host cell surface. Additionally, the ligand-binding preferences can promote the host cells’ infection with specific tissue tropism [81]. The previous studies revealed that brain infection with human CoVs is predominantly caused by virus migration to the cerebral microcirculation via ACE2-expressing endothelial cells. It has also suggested that the S protein of human CoVs may interact with additional neurotropism-related receptors in the brain when the ACE2 expression is at a low level [82]. In the context of sialome importance, the propagation of sialic acid-recognizing human CoVs can be additionally promoted by axonal transport. It has been shown that NTD of S proteins recognizes predominantly 9-O-acetylated sialic acids [83,84]. According to the study by Dube et al., the neuron-to-neuron transmission of human O43-CoV is mediated by axonal transport that increases its neuropathogenic potential [85]. The last study by Nguyen revealed that SARS-CoV-2 recognizes ganglioside GM1 containing one residue of α2.3-linked N-acetyl-neuraminic acid in the saccharide chain attached to ceramide [15]. As shown, ganglioside GM1 represents the majority (28%) of all gangliosides expressed in the adult brain and the highest level when compared to other organs [20]. Given its broad distribution and role in essential neuronal processes, GM1 may determine SARS-CoV-2 neurotropism. Moreover, the recent structural studies showed that two monosialylated molecules of GM1 form a complex with the GBD of S protein and thereby boost the interaction between SARS-CoV-2 and ACE2 [79,80]. Besides the invasion promoting the role of sialic acids, the sialylation of ACE2 seems to play a protective function against SARS-CoV-2 infection [86]. The experimental studies with cultured cell lines of various histological origins found that digestion of ACE2-expressing cells with neuraminidase increased the interplay between ACE2 and SARS-CoV-2, and thereby the infective potential [87].

Besides the sialic acid on the host’s glycocalyx, sialoglycans expressed by SARS-CoV-2 may act as potential factors that mediate virus attachment to host cells. It has been shown that human antigen-presenting cells (APC) can participate in antiviral immunity, but also boost viral spread via Siglec-1 (CD169) [88]. Siglec-1 is a type I transmembrane lectin that interacts with sialylated glycoconjugates that contain α2–3 linked N-acetylneuraminic acid (Neu5Ac). The structural studies showed that extracellular domains of Siglec-1 are able to recognize and bind different viruses, including HIV, Ebola and SARS-CoV-2, through viral membrane gangliosides (Figure 1C). In cases of endocytosis, the viral particles are accumulated in the storage compartment and then released to infect the host cells [89]. Perez-Zsolt et al., the SARS-CoV-2 infected APCs showed higher trans-infectivity towards ACE2-expressing cells than ACE2 lacking cells. The importance of this phenomenon in the SARS-CoV-2 induced neuropathology is limited by the expression of Siglec-1 [90]. In the human healthy brain, the distribution analysis of Siglec-1 confirmed the high level of positive perivascular and choroid plexus macrophages, whereas microglial cells are Siglec-1 negative [91]. Pellegrini et al. suggest that the strong SARS-CoV-2 tropism leads to damage to the choroid plexus epithelium, resulting in the entry of immune cells and cytokines into the cerebrospinal fluid and the brain [92]. The animal studies showed that the insult to the brain induces accumulation of Siglec-1-positive cells in the damaged area; however, the role of Siglec-1-expressing immune cells in the progression of neuropathology requires further studies [91].

### 4.2. Sialic Acids—Indicators of SARS-CoV-2-Induced Pathology in the Brain?

As mentioned, the unique structural properties of sialic acids allow them to enact pivotal functions in cell biology. The proper sialylation pattern and expression of sialylated glycoconjugates depends on the enzymatic machinery that regulates sialic acid homeostasis [93]. The aberrant sialome engineering disturbs cellular interactions, the conformation of cell membrane glycoconjugates and masking effects of antigenic determinants. In the brain, it is of particular importance in the context of synapse formation, long-term potentiation (LTP), long-term depression (LTD), tissue architecture controlling as well as the cell–cell and cell–microenvironment communication that underlie development, cognition, regeneration and immunity [71,94,95]. The unbalanced sialylation and desialylation have been confirmed in various CNS pathologies including malignancies, inflammation and neurodegeneration. Interestingly, there is increasing evidence that sialylation pattern is a sensitive indicator of pharmacological therapies and exposure to degenerative factors. As shown, changes in both cell membrane-bound sialoglycans and total serum sialic acids have been considered clinically significant as prognostic markers in cancers [96,97].

**Figure 2:**
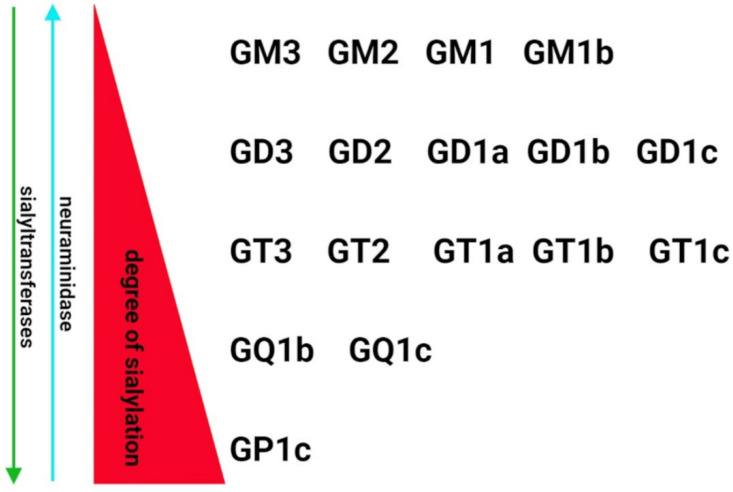
Sialylation pattern of representative human gangliosides. The degree of sialylation is controlled sialyltransferases and neuraminidase that mediate synthesis and degradation of gangliosides, respectively. The structure of the sialylated oligosaccharidic chain undergoes modulation by sialic acid-metabolizing enzymes that can be altered in response to cell activation or pathological stimuli.

Despite growing evidence on the role of sialic acids in SARS-CoV-2 infectivity, little is known about the relationship between SARS-CoV-2-induced pathology and the level of sialylation and changes to the sialic acids. Due to the lack of experimental data and molecular biology-based clinical investigations, the conclusions in this field remain speculative. Hyperactivation of the immune system is the common mechanism of nervous system degeneration in response to viral infection [98]. An excessive increase in the expression of proinflammatory cytokines is closely related to hyperinflammation in the respiratory tract that leads to uncontrolled systemic immune response and multiple organ dysfunctions. Due to the neurotropic features and transinfectious activity, SARS-CoV-2 presents the ability to infect macrophages, microglia and astrocytes that can be activated to produce and secrete an enhanced amount of inflammatory factors, including IL-6, IL-1β, IL-12, IL-15 and TNFα [99,100,101]. In response to the overproduction of immune cells and their compounds, the BBB become damaged and multiple multifocal lesions within the brain stem, cerebellum and cerebral white matter are observed [102,103,104]. The clinical statistics indicates that COVID-19-related hypoxic and metabolic encephalopathy as well as encephalitis are less frequent; however, the devastating effects of SARS-CoV-2 in the CNS are a major health-threatening consequence [105,106]. Despite the molecular mechanisms of neuropathology related to COVID-19 have not been fully characterized, there are a growing number of studies on glycocalyx reconstruction in response to inflammatory stimuli [107]. The animal models of inflammation showed alterations in α2.3-, α2.6- and α2.8-sialic acid glycotopes as a consequence of immune activation. In the chemically induced model of inflammation, an increase in the sialylation of serum glycoproteins was accompanied by upregulated expression of sialyltransferases [108,109]. However, some data demonstrated the opposite effects of lipopolysaccharide on sialoglycans in the brain. In the adult rats, the systemic inflammation resulted in an increased content of sialic acids in glycosylation patterns in most of the brain structures [107]. In contrast, a sustained reduction in the sialylation of cerebral glycoproteins was detected following postnatal inflammatory exposure. It was correlated with increased neuraminidase 1 (Neu1) mRNA and its activity in perinatal infection exposure (Figure 1B) [110]. The study by Timur et al. suggest that neuroinflammation is closely related to the impaired catabolism of gangliosides, including GM1 and GD1a, due to decreased expression of neuraminidase 4 (Neu4) [111]. As mentioned in Section 2, the brain gangliosides contain the majority of tissue glycoconjugate-bound sialic acid. The expression and structure of major gangliosides are regulated by the internal or terminal incorporation of sialic acid into the neutral glycan chain featured by the same sugar sequence (Galβ1-3GalNAcβ1-4Galβ1-4Glcβ1-1Cer) [20] (Figure 2).

Indeed, the attachment of the following sialic acid residues to the basal ganglioside GM3 forms structurally specific derivatives that can reflect the metabolism of gangliosides in response to pathological stimuli. The clinical observation revealed a significant reduction in total ganglioside amounts in the temporal cortex, hippocampus and white matter of AD patients [94]. As shown, the affected brain areas manifested elevated levels of GM3, GM2, GM1 and GT1a, and decreased amounts of GD1b and GT1b that may suggest differences in the sialylation/desialylation balance in neurodegenerative processes [94]. Moreover, an increased level of sialylated GM1, GM2, and GM3 has been observed in hypoxia, the major cause of brain damage in COVID-19, that disturbs the sialylation of the glycocalyx and induces inflammation and oxidative stress in brain regions that manifest neurodegeneration [112,113,114]. It is of particular importance as a higher risk factor for Alzheimer’s disease and other neurodegenerative changes [115].

**Figure 3:**
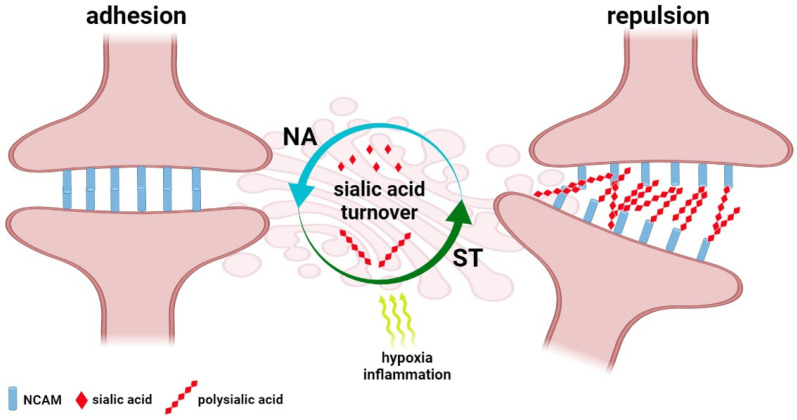
The regulatory function of polysialic acid turnover in the brain. Polysialic acid (PSA) is a linear homopolymer of a 2,8-linked sialic acid that forms a sugar chain with steric properties due to a high density of negative charges. At the posttranslational level, NCAMs are modified through attachment of PSA in the Golgi compartment by typical mechanism of N-linked core glycosylation. In result, the 2,8-linked sialic acid prevents homophilic interactions between synaptic NCAMs and their heterophilic interactions with homologous ligands. These alteration in the brain are accompanied by an increase in the number of PSA-NCAM immunoreactive neurons that results in the modification of synapse density in response to degenerative factors, including inflammation and hypoxia.

Besides the devastating, COVID-19-related consequences for the brain, subtle cognitive and psychological symptoms are also observed in SARS-CoV-2-infected individuals. In most cases, the post-COVID-19 alterations are characterized by confusion, lapses of memory, and a lack of focus and mental clarity, whereas cognitive impairment of varying intensity has been confirmed by memory tests in patients of intensive care units hospitalized due to acute respiratory failure and shock [116]. Moreover, the assessment of the prevalence of psychiatric signs following COVID-19 showed the particular importance of infection in the development of depressive disorder. Statistical analysis of depression incidences in COVID-19-infected individuals that were clinically monitored within the Long-Term Neuropsychiatric Disorder in COVID-19 Project confirmed the close relationship between the infection process and gender, previous pulmonary and mental dysfunctions and development of depression [117]. Despite the novel directions in the therapy and prevention of CNS dysfunction in COVID-19 being developed, the sialic acid-based mechanisms that underlie their progression are still unclear [118]. However, sialome seems to be dependent on multiple body-associated factors, including hormonal set and physiological status, that contribute to altered sialylation and partly reflect the prevalence and severity of COVID-19 in sex and age-dependent manner [119]. Brain plasticity, including neurogenesis and reorganization of natural pathways, is a precise but sensitive mechanism that underlies learning and memory [120,121]. The long-COVID-related cognitive dysfunction, called “brain fog”, is not featured as dementia symptoms and structural damage to the brain; however, quantitative and qualitative changes in sialylation patterns within synaptic connections may affect the synaptic architecture and cause reversible cognitive deficits. Similarly, the neuronal plasticity theory of depression focuses on alterations in dendrites and spine density that lead to impaired balance of excitatory and inhibitory neurotransmission [122]. As shown, changes in NCAM and PSA-NCAM expression are the main regulatory mechanism of adhesion and repulsion within the synapse [123] (Figure 3).

Molecular analysis of hippocampal tissue showed an enhanced level of PSA–NCAM that was correlated with severe neurodegenerative alterations expressed as tissue remodelling, structural changes of cells and neuronal loss [124]. It is of particular importance in the field of depression since the PSA–NCAM-regulated plasticity is known as a complex mechanism that bridges the monoamine, neurogenic, and neurotrophic theories of depression [122]. Exposures to anti- and pro-inflammatory cytokines, chemokines and ROS are known to exert effects on basic steps of neurogenesis, including neural progenitor cell proliferation, migration, differentiation, survival and incorporation of newly formed neurons into the CNS. These processes can be reinforced by mild acute inflammation, but chronic proinflammatory stimulation injures neurogenesis [125,126]. In the field of glycobiology, cognitive functions are closely dependent on the modification of expression and function of synaptic proteins and gangliosides. Despite the fact that the SARS-CoV-2-induced changes in the host’s glycome are not fully understood, the pathogen-related structural changes to cellular glycans have been detected in multiple animal models. According to Rehan, the Toxoplasma gondii-induced depressive-like behaviours are closely correlated with overexpression of N-glycans terminated with N-glycolylneuraminic acid (Neu5Gc) and N-acetylneuraminic acid (Neu5Ac). Therefore, these sialylated structures have been proposed as novel biomarkers of sickness/depressive-like behaviours [73]. There is multiple evidence for ST3Gal3, ST8Sia2 and ST8Sia4 as crucial modulators of sialoglycans recruited in synaptic plasticity [20,127]. Indeed, variations in the enzymatic machinery of sialylation are linked to reduced spatial working memory. The experimental data revealed that synaptic PSA-NCAMs are the main regulators of synaptic plasticity in the hippocampus and thereby stabilize its function in cognitive processes [128]. In contrast, the enzymatic removal of PSA leads to LTP and LTD disturbances in the hippocampus and memory loss, hypoxia and inflammation induce enhancement of PSA–NCAM that correlates with learning defects [129,130,131,132]. The quantitative and qualitative changes in the polymerisation of polysialic acid have been described in psychiatric disturbances and neurodegenerative processes and their critical importance has been confirmed for healthy brain function. This phenomenon closely depends on both hypersialylation and hyposalivation, which alter synaptic function based on signalling pathways recruiting neurotransmitters, e.g., dopamine as well as growth factors, including brain-derived neuronal factor (BDNF) and fibroblast growth factor (FGF). Interestingly, the binding affinity of dopamine, FGF and BDNF by polysialic acid is controlled by ST8Sia2, whereas its dysfunction results in the misguidance of synapses in selective brain areas and contributed to various morphological, cognitive, and emotional deficits [133]. In addition, COVID-19 related emotional dysfunctions, including stress, anxiety and sleep deprivation increase PSA–NCAM immunoreactivity in brain regions [131,134,135]. These alterations can be accompanied by the loss of spatial learning abilities; however, the participation of polysialic acid in the endogenous mechanisms of repair should be also considered [136].

Given the importance of sialic acid in the dual mechanism of SARS-CoV-2 attachment and entrance, several sialylated ligands have been described as crucial in the autoimmunity of the brain. As mentioned previously, the viral S protein recognizes specific sugar residues of gangliosides and facilitates anchoring in the cell membrane. However, the sialylated cell membrane compounds of the host demonstrate the high similarity with a viral sialic acid repertoire that underlies the molecular mimicry (Figure 1D). According to Dalakas, the cross-reaction between S protein and GM1 and ganglioside dimers in neuronal cells trigger neuropathies of different degrees of involvement of motor and sensory cells, myelin sheath and axons [137]. This phenomenon has been also observed in infections caused by *Zika virus* and *Campylobacter jejuni* that interact with glycolipids widely expressed on peripheral nerve surfaces [138,139]. Interestingly, these processes can be blocked by chloroquine due to the blocking effect on cellular glycocalyx; however, it has been not used as therapeutic management in COVID-19 [79]. The multiple neurological observations indicate that inflammatory neuropathy, including Guillain-Barré syndrome (GBS), is the late effect of SARS-CoV-2 neurovirulence [140,141]. In response to the infection, the IgM and IgG against multiple gangliosides are produced by the immune system. In the post-COVID-19 cases, multiple antibodies against neuronal gangliosides have been detected as associated with inflammatory neuropathy. Several gangliosides, including disialylated GD1b, GQ1b and GT1b, as well as combined GM1 and GM2, serve as antigens for IgG and IgM antibodies that contribute to clinical symptoms in the CNS [140]. The observations by Dalakas et al. indicate that interaction of IgM antibodies with GM1 or GD1b gangliosides expressed on motor and dorsal root ganglionic neurons, respectively, results in sensory ataxic neuropathy. It should be stressed that changes in neuronal sialylation patterns in GBS have not been described; however, quantitative and qualitative alterations in antigen–antibody interaction can be used as prognostic markers. Indeed, the clinical improvement in symptoms has been found to be correlated with the decay of antibodies directed against several neuronal gangliosides of the host, including GM1, GD1a, GT1a, GM2, and GQ1b [140]. Moreover, the repair processes in the demyelinating form of GBS can be facilitated by the reduction of polysialic acids that act as major remyelination inhibitors [142].

### 4.3. Sialoglycans-Siglec Axis—Regulatory Mechanism of Clinical Course in SARS-CoV-2 Invasion in the Brain?

As mentioned in Section 2, the sialic acid–Siglec axis is a part of the signalling system that regulates the function of immune cells. Interestingly, several viral classes utilize Siglec to facilitate fusion on antigen-presenting cells and mediate transfection to the other target cells in trans infection. The role of Siglec-1 has been described in multiple viral infections; however, the interaction between CD33-related Siglecs and SARS-CoV-2 remains still an open question. However, given that the sialylated residues are broadly expressed in SARS-CoV-2, the modulation of the hosts’ immune response via Siglecs is highly possible (Figure 1E). It is of particular importance in the context of the hosts’ phenotype-dependent Siglecs expression profile in the resident and infiltrating immune cell populations. In the human brain, the innate immune response in microglia is mainly controlled by the paired Siglec-11 and Siglec-16 receptors that trigger inhibitory and activatory signalling, respectively, and counterbalance the state of cellular activation in response to the binding of α2.8-linked sialic acids. Siglec-11 and Siglec-16 are featured by 99% of sequence identity at the extracellular domains; however, the intracellular signalling is transduced by the opposite pathways based on immunoreceptor tyrosine-based inhibition motif (ITIM) and immunoreceptor tyrosine-based activation motif (ITAM), respectively [143]. The importance of Siglec-11 in the regulation of brain immunity has been described in malignancy and bacterial infections. As shown the neuronal PSA-NCAMs alleviate the microglia-induced neurotoxicity, whereas their enhanced expression in high-grade brain tumours promotes the antigens hiding and participates in “Off” signalling via Siglec-11 [144]. The “Off” signalling is a part of communication between phagocyting cells and neurons that result in the inhibition of phagocytic activity under normal conditions. However, the desialylation of neuronal surfaces disturbs the inhibitory signalling pathway and simultaneously activates the complementary system that promotes phagocytosis and neuronal loss. Additionally, Siglec-11 is a main player in molecular mimicry, which promotes the invasion of neonatal brains by *Escherichia coli K1* via recognition and binding of capsular polymers of α2.8-linked sialic acids known to be identical to the human PSA [145]. Additionally, the immunoregulatory function of Siglecs in the CNS is not only restricted to microglial cells; however, the broad distribution of several Siglecs in the subsets of infiltrating immune cells suggests their role in the brain pathology. Similarly to paired Siglec-11/16, Siglec-5 and Siglec-14 on the infiltrating monocytes, macrophages and neutrophils, downregulate or upregulate leucine-rich repeat and pyrin domain-containing-3 (NLRP-3) inflammasome activity, respectively, using SHP-2-based inhibitory or DAP12-based activator signal transduction [146]. Based on the previous observations, Murch hypothesizes that SARS-CoV-2, similarly to SARS-CoV, affects the paired Siglec-5/14 via sialylated secreted glycoproteins (SGP) [147]. In line, the interplay between sialoglycans and activatory counterpart of paired Siglecs can be considered as a part of cytokine storm machinery [18]. Interestingly, the preferable promotion of paired Siglecs counterparts is linked to high genetic variability and seem to be correlated with the clinical outcome of the immune response. As shown, individuals with predominant expression of Siglec-14 develop a potentiated immune response toward the pathogens, while the loss of SIGLEC-14 gene in Siglec-5 expressing patients is accompanied by immunosuppression [148,149,150,151]. Therefore, the opposite effects of Siglec-based regulation of immunity and variable distribution of paired Siglecs in individuals can partially explain the specific features and demography-dependent differences in the clinical course of SARS-CoV-2 invasion in the CNS, and act as a predictive factor of severe course of infection.

## 5. Conclusions

In this brief review, we have considered the importance of sialoglycans in the mechanism and consequences of SARS-CoV-2 invasion in the CNS. The progress in the field of virology and glycobiology as well as multiple clinical observations help to understand how sialic acids determine the virus attachment, its destination and route in the brain. The available clinical data suggest that neuronal sialoglycans participate in the acute phase of infection and lead to the development of neuronal dysfunctions in the chronic phase caused by SARS-CoV-2. Given the broad expression of sialome compounds of cell membranes and their role in the structure and neuronal function, modulation of virus-sialic acid interplay seems to present also preventive and therapeutic potential in the future management of SARS-CoV-2 infection and mitigate the COVID-19-related pathology in the CNS.
